# A stakeholder-driven agenda for advancing the science and practice of scale-up and spread in health

**DOI:** 10.1186/1748-5908-7-118

**Published:** 2012-12-06

**Authors:** Wynne E Norton, C Joseph McCannon, Marie W Schall, Brian S Mittman

**Affiliations:** 1 Department of Health Behavior, School of Public Health, University of Alabama at Birmingham, 1665 University Boulevard, Birmingham, AL, 35294, USA; 2 Institute for Healthcare Improvement, 20 University Road, Cambridge, MA, 02138, USA; 3 VA Center for Implementation Practice and Research Support, VA Greater Los Angeles Healthcare System, 16111 Plummer Street, North Hills, CA, 91343, USA

**Keywords:** Scale-up, Spread, Healthcare, Public health, Improvement, Implementation, Dissemination, Health systems research, Healthcare delivery

## Abstract

**Background:**

Although significant advances have been made in implementation science, comparatively less attention has been paid to broader scale-up and spread of effective health programs at the regional, national, or international level. To address this gap in research, practice and policy attention, representatives from key stakeholder groups launched an initiative to identify gaps and stimulate additional interest and activity in scale-up and spread of effective health programs. We describe the background and motivation for this initiative and the content, process, and outcomes of two main phases comprising the core of the initiative: a state-of-the-art conference to develop recommendations for advancing scale-up and spread and a follow-up activity to operationalize and prioritize the recommendations. The conference was held in Washington, D.C. during July 2010 and attended by 100 representatives from research, practice, policy, public health, healthcare, and international health communities; the follow-up activity was conducted remotely the following year.

**Discussion:**

Conference attendees identified and prioritized five recommendations (and corresponding sub-recommendations) for advancing scale-up and spread in health: increase awareness, facilitate information exchange, develop new methods, apply new approaches for evaluation, and expand capacity. In the follow-up activity, ‘develop new methods’ was rated as most important recommendation; expanding capacity was rated as least important, although differences were relatively minor.

**Summary:**

Based on the results of these efforts, we discuss priority activities that are needed to advance research, practice and policy to accelerate the scale-up and spread of effective health programs.

## Background

Recognizing the need for better methods to accelerate adoption of effective health practices and programs, researchers and funding agencies have expanded work in implementation science and the related disciplines of improvement science [1] and health systems and delivery research [2]. Generally speaking, these fields aim to identify barriers and facilitators to the adoption and use of effective practices and programs, and develop, test, and refine strategies for bridging the research-to-practice gap [1,3-5].

To date, however, most implementation studies have been conducted in relatively small- to moderately-sized samples of institutions, delivery systems, agencies, and/or communities [6-9]. Although studies in small samples provide useful insights regarding local barriers and facilitators to adoption, the relevance of these findings for efforts to achieve large-scale adoption (*i.e.*, scale-up or spread) in hundreds or thousands of institutions or communities is limited. Numerous practice-based efforts to scale-up and spread evidence-based health programs have been documented (although primarily in developing countries), but this work often does not employ theory-based, rigorous scientific approaches for studying scale-up processes, and thus offers limited evidence and guidance for improving future scale-up efforts. The terms ‘scale-up’ and ‘spread’ lack accepted, universal definitions [3,10-16]; we use the terms interchangeably and define scale-up and spread as ‘deliberate efforts to increase the impact of innovations successfully tested in pilot or experimental projects so as to benefit more people and to foster policy and program development on a lasting basis’ (p. viii) [17]. Others use the term ‘going to scale’ when at least 60% of the target population that could potentially benefit from the program receives it [18,19]. Development and use of the terms scale-up and spread are noted elsewhere [10,12,20].

Although limited, research interest in scale-up and spread is increasing. Much of this work has been conceptual or descriptive: developing frameworks and models for scale-up and spread [11,15,17,21-26]; discussing key issues in scale-up and spread [10,20]; and describing strategies for achieving scale-up and spread [13,14,27-29]. Furthermore, much of this work is narrowly focused, occurring in silos delineated by country, care setting, and/or health domain. There are few opportunities for cross-fertilization of ideas among researchers, practitioners, and policymakers engaged in scale-up and spread activities, and relatively few funding opportunities for research or practice efforts.

Responding to these challenges and the need for expanded research, practice, and policy to ensure that effective programs achieve impact on health at the population level, we launched a multi-stakeholder initiative to increase awareness and to identify specific actions needed to expand scale-up activity in health. The initiative was envisioned and launched during an informal, 30-person working dinner meeting held in junction with a panel session at the 2^nd^ Annual National Institutes of Health Conference on the Science of Dissemination and Implementation (2009). The dinner attendees proposed a state-of-the-art/agenda-setting conference involving approximately 100 U.S. and international representatives from research, practice, and policy in healthcare and public health, which we conducted during July 2010. The conference generated specific recommendations for actions needed to facilitate enhanced interest and activity in scale-up. A follow-up activity was conducted during Fall 2011 to prioritize and operationalize the recommendations. This article describes the methods and findings from the conference and the follow-up prioritization activity.

### State-of-the-art conference

The Conference to Advance the Science and Practice of Scale-up and Spread of Effective Health Programs in Healthcare and Public Health (hereafter noted as ‘the conference’) was held in Washington, DC from July 6-8, 2010. The conference was organized by representatives from the Institute for Healthcare Improvement (McCannon, PI), the University of Alabama at Birmingham School of Public Health (Norton), and the US Department of Veterans Affairs Quality Enhancement Research Initiative (Mittman). Approximately 100 individuals were invited to attend the conference, reflecting a purposeful mix from the research, practitioner, policymaker, public health, healthcare, U.S., and international communities (see Table 1 for represented agencies, organizations, and institutions). Support for the conference was provided by the U.S. Agency for Healthcare Research and Quality, The Commonwealth Fund and the Department of Veterans Affairs, with additional funding from The John A. Hartford Foundation and The Patrick and Catherine Weldon Donaghue Medical Research Foundation.

**Table 1:** Organizations, institutions, and agencies represented at the conference

	
American Board of Internal Medicine Foundation	National Implementation Research Network
Agency for Healthcare Research and Quality	National Institute of Child Health and Human Development
Bill and Melinda Gates Foundation	National Institute on Aging
Blue Cross Blue Shield	National Institute of Mental Health
CAPTURE Project	Oregon Social Learning Center
Centers for Disease Controland Prevention	The Patrick and Catherine Donaghue Medical Research
Center to Advance Palliative Care	Pennsylvania State University
Canadian Institutes of Health Research	Project Health
Centers for Medicare & Medicaid Services	VA Quality Enhancement Research Initiative
Common Ground	Robert Wood Johnson Foundation
Common Knowledge Associates	Stanford University
The Commonwealth Fund	Texas Health Science Center
Dana-Farber Cancer Institute	Transtria
Dimagi, Inc.	TRICARE Management Activity
Duke University	Universite Laval
ExpandNet	University of Alabama at Birmingham
Georgetown University	University of California, Los Angeles
Harvard University	University of California, San Francisco
Health Research and Educational Trust	University of Connecticut
Health Partners Research Foundation	Univ. of Medicine/Dentistry of New Jersey
Health Resources and Services Administration	University of Michigan
Institute for Healthcare Improvement	United States Agency for International Development
Iowa Health System	University of North Carolina at Chapel Hill
The John A. Hartford Foundation	University of Pennsylvania
Johns Hopkins University	University of Washington
Kaiser Permanente	University of Wisconsin
Karolinska Institute	University Research Co., LLC
McKinsey and Company	U.S. Army Medical Department
MedPAC	US Department of Education
Michigan Heath and Hospital Association	Washington University in St. Louis
National Committee for Quality Assurance	Yale University

The conference format was based largely on the VA’s state-of-the-art conference model [30,31]. The first full meeting day began with an overview presentation by the conference organizers, brief presentations by the authors of four commissioned papers [16,27,32,33], and a summary of objectives for the working groups. Five working groups, each comprised of approximately 15-20 individuals representing a mix of researchers, practitioners, and policymakers, were created prior to the meeting. Each working group was charged with three main tasks to accomplish over the next day-and-a-half: envision and describe an ideal system for scale-up; identify gaps between the current and future envisioned state; and develop recommendations for action to close the gaps. Working groups presented summaries of their recommendations to the broader group on the second full day of the conference, followed by a discussion of next steps.

### Follow-up activity

We re-engaged attendees one year after the conference to prioritize and operationalize the conference recommendations. The core conference planning group first refined and summarized the conference recommendations, producing five broad summary recommendations. We then drafted sub-recommendations describing specific actions to operationalize each of the five summary recommendations. Next, we invited 126 individuals to complete an online survey to rate the importance of each recommendation and sub-recommendation for advancing scale-up effective health programs (1 = Unimportant, 5 = Very Important). The 126 individuals included attendees from the conference as well as approximately 25 other individuals identified by colleague referrals. Of the 126 eligible individuals, 49 (39%) completed the survey.

## Discussion

The five major conference recommendations, their associated sub-recommendation ‘action items,’ and participant importance ratings are listed in Table 2. Below, we describe each recommendation and its key sub-recommendations.

**Table 2:** Recommendations, sub-recommendations, and ratings

**Recommendation**	**M (*SD*)**
**1. Increase awareness of the need for greater attention and activity in scale-up and spread, including research, practice and policy activity.**	4.31 (0.78)
1.1 Educate healthcare and public health agencies and professionals regarding the need for explicit, pro-active initiatives to achieve scale-up and spread of effective health programs.	4.26 (0.90)
1.2 Provide professional and monetary incentives for researchers, practitioners, and policymakers to pursue scale-up/spread activities.	4.00 (1.08)
1.3 Convene an Institute of Medicine/Canadian Academy of Health Sciences (or other entity) committee to assess the current status of research, practice and policy activities in scale-up and spread.	3.96 (1.00)
1.4 Convene a multi-stakeholder group to create an overarching blueprint for expanding interest and activity in scale-up/spread research, practice and policy.	3.56 (1.06)
1.5 Educate the general public to enhance pull for scale-up/spread of effective programs.	2.88 (1.00)
**2. Facilitate better information exchange, collaboration and use of existing knowledge regarding scale-up and spread.**	4.28 (0.73)
2.1 Synthesize existing knowledge (and incorporate new knowledge as developed) to create practical guidance for scale-up and spread practice and policy.	4.34 (0.85)
2.2 Develop and facilitate online communities of practice in scale-up and spread research, practice and policy that are specific to stakeholder groups, health areas, or delivery settings.	3.75 (0.78)
2.3 Increase clinical and related data sharing among all major health agencies (*e.g.*, Centers for Medicare & Medicaid Services, Health Resources and Services Administration, Centers for Disease Control and Prevention, etc.).	3.65 (1.06)
2.4 Create a Center for Scale-up/Spread comprising multiple federal and private agency representatives to increase collaboration and progress in advancing scale-up and spread knowledge and activity.	3.57 (1.13)
2.5 Create an online, interactive learning network for all types of scale-up/spread stakeholders in health care and public health to engage with one another.	3.55 (0.85)
**3. Develop, evaluate and refine innovative scale-up and spread methods, including novel incentives and pull strategies.**	4.56 (0.64)
3.1 Develop new funding announcements to conduct ‘embedded research’ on practice- and policy-led scale-up/spread initiatives (*e.g.*, natural experiments).	4.53 (0.68)
3.2 Develop new funding programs to support investigator-initiated scale-up/spread research.	4.44 (0.70)
3.3 Identify and strengthen existing research programs studying scale-up and spread.	4.18 (0.74)
3.4 Develop taxonomy of scale-up/spread strategies and related concepts to describe scale-up/spread approaches and factors.	4.12 (0.93)
**4. Develop and apply new approaches for evaluation of scale-up and spread.**	4.46 (0.71)
4.1 Develop, evaluate and implement practical measures of spread of health practices and programs to facilitate improved research and enhanced tracking of scale-up/spread progress.	4.35 (0.66)
4.2 Convene a planning group to advance the idea of embedded evaluation to generate more knowledge about scale-up and spread in the context of ongoing policy/practice initiatives.	4.11 (0.93)
4.3 Convene consensus groups and stimulate research to develop innovative approaches for studying scale-up and spread processes and mechanisms (and their determinants) to better understand how, when, where, and why scale-up and spread strategies operate.	4.08 (0.87)
4.4 Convene a planning group to address research and evaluation barriers related to Institutional Review Board regulations and other challenges.	3.80 (0.89)
**5. Expand capacity for scale-up and spread policy, practice and research.**	4.18 (0.83)
5.1 Identify funding sources to support workforce preparation activities, including course and curriculum development, credentialing, training, and mentoring.	4.17 (1.06)
5.2 Identify experts in scale-up and spread to serve as mentors for new scale-up/spread research, practice and policy experts.	4.02 (0.83)
5.3 Develop courses on scale-up/spread in health care and public health for inclusion in established health-related degree programs (*e.g.*, MD, RN, MSW, MPH, MS, PhD).	4.01 (0.95)
5.4 Develop non-degree courses and training programs for researchers, practitioners, and policymakers focused specifically on scale-up and spread, including courses addressing the role of data in guiding and supporting scale-up and spread efforts.	3.85 (0.98)
5.5 Convene a group to develop credentialing requirements and programs for individuals engaged in scale-up/spread initiatives.	2.83 (1.16)

Note. Response options include 1 = Unimportant, 2 = Low Importance, 3 = Moderately Important, 4 = Important, 5 = Very Important. Higher scores indicate greater importance of recommendation. Within each of the five major recommendations, sub-recommendations are listed from highest score (*i.e.*, greatest importance) to lowest score (*i.e.*, lowest importance).

### Recommendation #1: Increase awareness of the need for greater attention and activity in scale-up, including research, practice, and policy activity

Stakeholders noted the need for a rich portfolio of follow-up activities to maintain engagement and enthusiasm and to stimulate interest and involvement by additional stakeholders. Although the multi-stakeholder conference itself was intended to stimulate greater interest and activity, the conference attendees and survey respondents recognized the need for sustained follow-up efforts. Highly-rated suggestions included targeted, intensive outreach and education of the need for focused attention on scale-up and spread, as well as financial incentives and support.

### Recommendation #2: Facilitate better information exchange, collaboration and use of existing knowledge

Stakeholders noted that a considerable amount of activity in scale-up research, practice, and policy is not widely known, and thus fails to achieve its full benefit. They recommended creating a database and related mechanisms (*e.g.*, email groups, conference calls, meetings) for tracking and sharing information regarding ongoing policy, practice, and research in scale-up. This system would facilitate increased communication and collaboration among key stakeholders, more rapid learning and progress, and greater efficiency in resource utilization. Additional recommendations included the development of practical summaries of existing knowledge and research results to facilitate their use and benefits.

### Recommendation #3: Develop, evaluate and refine innovative scale-up and spread methods, including novel incentives and ‘pull’ strategies

Stakeholders recognized the need to further test and refine existing methods for effectively scaling-up health practices and programs, including a better understanding of when, where, and how particular methods are more or less effective. For example, research is needed to assess the effectiveness of methods to spread simple practices in specific settings (*e.g.*, hospital intensive care units) compared to methods to scale-up complex interventions across larger, interdependent systems (*e.g.*, chronic disease management and prevention programs requiring collaboration across hospitals, ambulatory care centers, and homes). Stakeholders also emphasized the need to better understand conditions under which sufficient demand (*i.e.*, ‘pull’) for scale-up of innovations will arise to complement or replace the supply-oriented (*i.e.*, ‘push’) approaches typically employed in small-scale, local implementation initiatives. Specific actions recommended to facilitate this knowledge production include the development of new funding opportunities for practice-based, practice-oriented research, the identification and expansion of existing programs supported by such research, and research efforts to develop taxonomies of scale-up strategies and factors influencing their effectiveness.

### Recommendation #4: Develop and apply new approaches for evaluation

Stakeholders noted the dominance of quantitative experimental approaches evaluating scale-up processes and strategies, and highlighted the need for more flexible studies involving real-time collection of both qualitative and quantitative data for use in guiding ongoing adaptations during the scale-up process. Stakeholders also highlighted: the need for increased observational research on naturally-occurring spread processes; the value of evaluation methods that allow for continuous learning and the need for increased efforts to understand the role of contextual influences and to understand mechanisms of effect of scale-up strategies; and methods suitable for studying complex adaptive systems and scale-up processes that do not progress in a linear manner. Specific actions recommended to facilitate progress in this area include the development of research and evaluation tools such as standardized measures of scale-up and spread, and the establishment of consensus groups to identify and promote innovative research approaches useful in observational studies and in research examining scale-up mechanisms and processes in addition to their outcomes and impacts.

### Recommendation #5: Expand capacity for scale-up policy, practice, and research

Stakeholders recognized that success in achieving these goals will require expanded human resources, capacity, and expertise. They suggested a series of activities to expand scale-up capacity, including the development of curricula and courses on scale-up and their inclusion in professional programs offered by schools of public health and programs training other health professionals. Additional recommended actions include recognition and rewards for researcher involvement in scale-up studies and in policy and practice efforts and increased funding to support these efforts. Stakeholders expressed the need for learning activities that link stakeholders together to share new concepts, critique ongoing scale-up activities and, through these interactions, enhance stakeholder skills and expertise and stimulate greater interest. Stakeholders also challenged the field to tackle projects on an unprecedented scale and at an unprecedented pace that would serve as powerful demonstrations of change, and subsequently bring more attention, legitimacy, and recognition to the field.

### Limitations

Limitations of the recommendations and corresponding sub-recommendations generated from the conference and follow-up activity should be noted. The recommendations resulted from open discussion and brainstorming, and are neither comprehensive nor exhaustive; we welcome additional suggestions and ‘action items’ for advancing the field. The recommendations and their priority ratings reflect the composition of conference and survey participants (*e.g.*, few representatives from low- and middle-income countries; few representatives from patient advocacy groups; overrepresentation of researchers), and thus might be biased and emphasize actions of interest to researchers and stakeholders in developed countries.

## Conclusions

In sum, the initiative described herein brought together expert researchers, practitioners, and policymakers to identify and prioritize next steps for advancing the field in a way that sought to minimize isolated projects conducted within individual silos, capitalize on the depth of knowledge and expertise available, and stimulate future activity. As an extension of the initiative, the authors are launching several follow-up activities to pursue these recommendations, including development of working groups charged with tackling the identified priority recommendations and sub-recommendations, delineated by a focus on research-based activities (*e.g.*, develop registry of scale-up/spread expert researchers and projects; develop list of specific research questions or goals that might be addressed through new funding programs) and practice-based activities (*e.g.*, providing peer consultation on the design and execution of emerging scale-up/spread projects; creating a mechanism to easily exchange information and provide support to scale-up/spread practitioners). The recommendations and sub-recommendations described herein are meant to specify actions to advance research, practice, and policy in scale-up and spread in health, and galvanize interest and effort in this area that are commensurate to its need.

## Competing interests

WEN is on the Editorial Board of *Implementation Science*. CJM is now at the Centers for Medicare & Medicaid Services; he has reviewed the material herein for factual accuracy but has not made substantive contribution since summarizing the meeting and its findings prior to leaving the IHI. MWS reports no conflicts of interest. BSM is Editor-in-Chief Emeritus of *Implementation Science*.

## Authors’ contributions

All authors contributed to the development, organization, and execution of the state-of-the-art conference. CJM wrote the first draft of the summary of the conference. BSM, WEN, and MWS were responsible for conducting the follow-up online rating study. WEN drafted the first version of the full meeting report; BSM and MWS revised the manuscript. CJM reviewed the revised manuscript for factual accuracy. All authors read and approved the final manuscript.
